# Supplementary figures and images for: A simple statistical model and physical device to estimate a woman-specific probability of skilled birth assistance and associated benefit of maternity waiting home stay
Source: PLoS One. 2025 May 19;20(5):e0302245. doi: 10.1371/journal.pone.0302245 (PMC12088025; doi:10.1371/journal.pone.0302245)

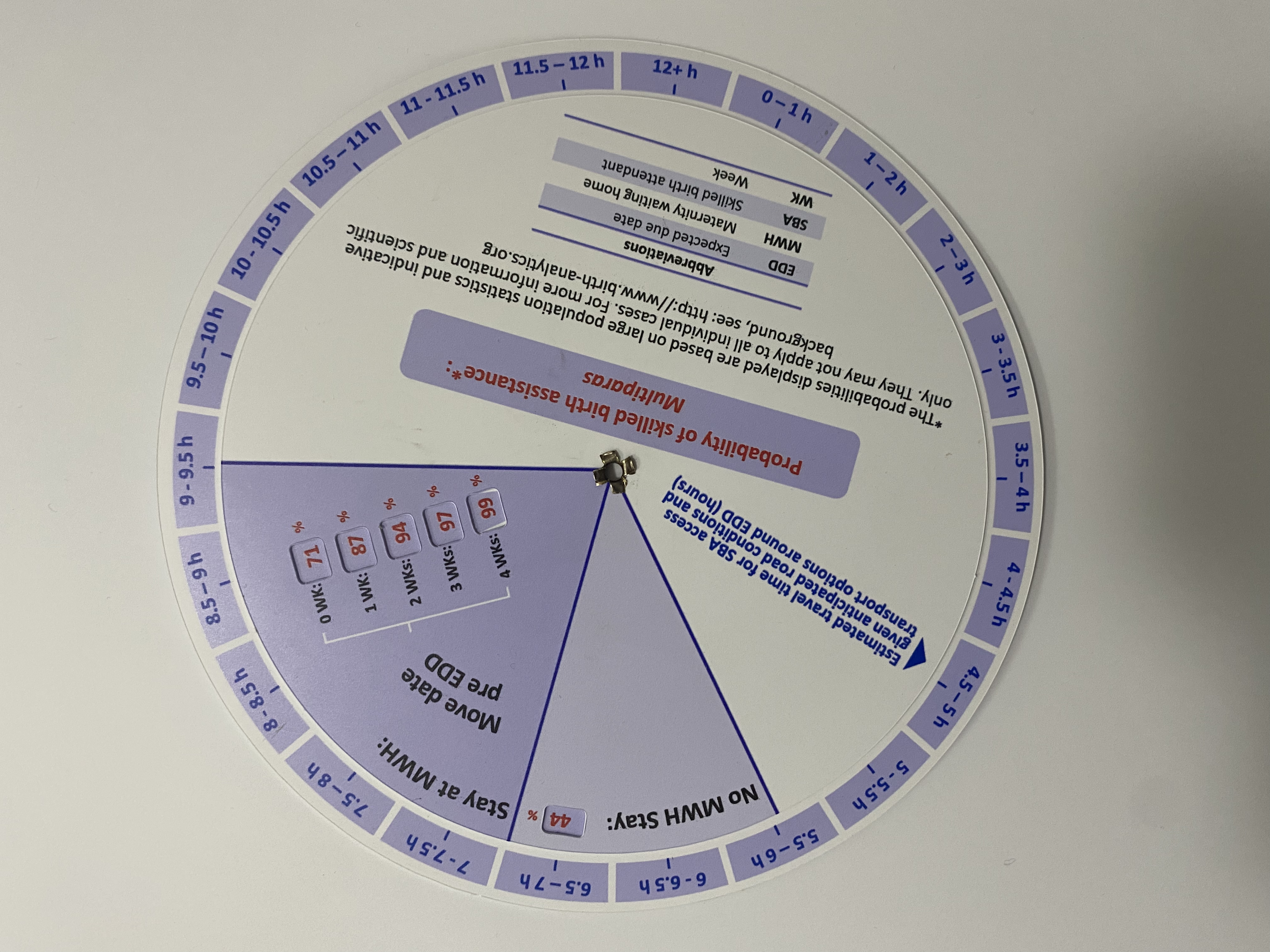

Supplement: S1 file — (JPG) [file pone.0302245.s001.jpg]

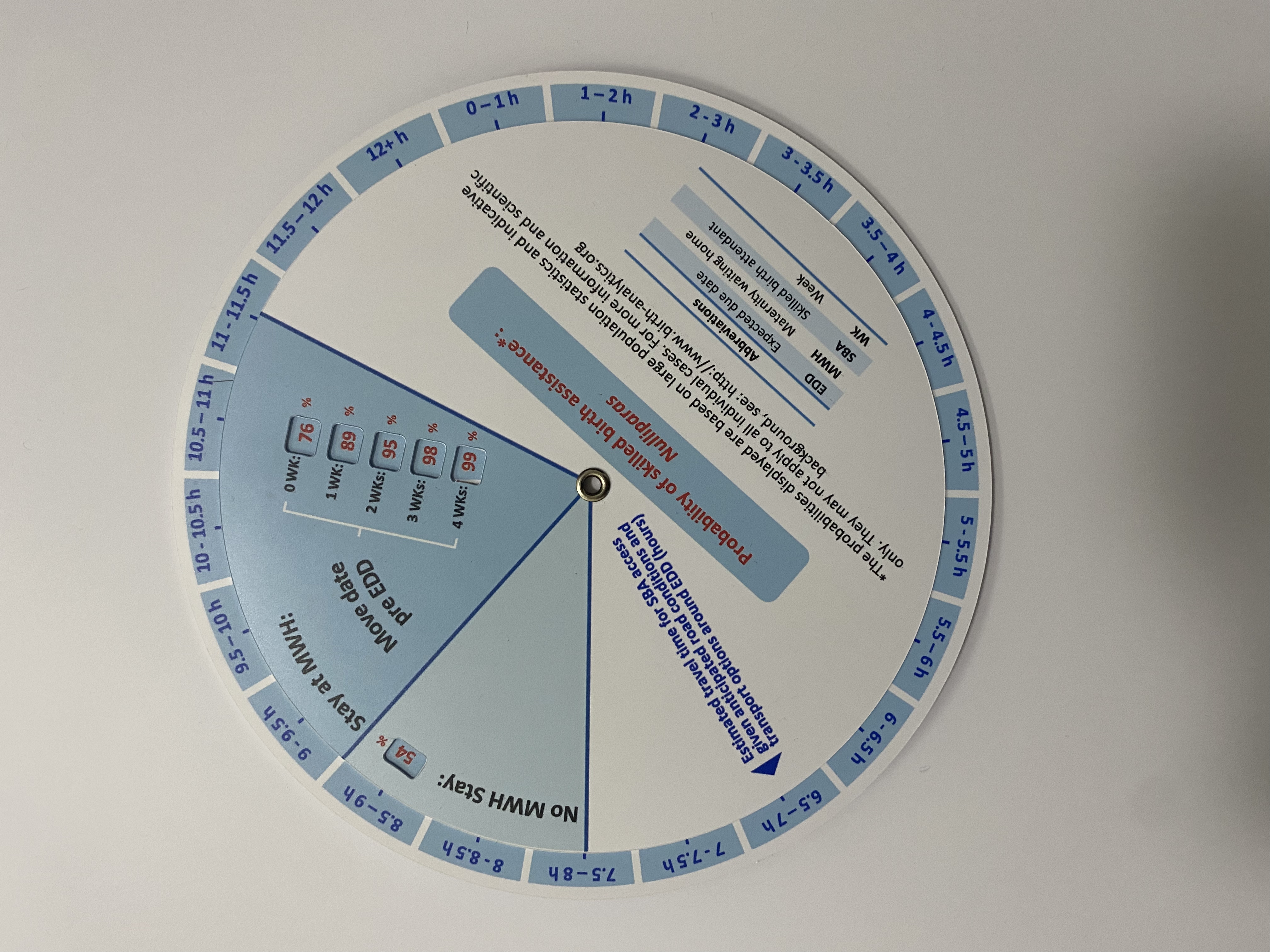

Supplement: S2 file — (JPG) [file pone.0302245.s002.jpg]
